# Acceptance of smart sensing: a barrier to implementation—results from a randomized controlled trial

**DOI:** 10.3389/fdgth.2023.1075266

**Published:** 2023-07-13

**Authors:** Yannik Terhorst, Nadine Weilbacher, Carolin Suda, Laura Simon, Eva-Maria Messner, Lasse Bosse Sander, Harald Baumeister

**Affiliations:** ^1^ Department of Clinical Psychology and Psychotherapy, Institute of Psychology and Education, University Ulm, Ulm, Germany; ^2^ Department of Rehabilitation Psychology and Psychotherapy, Institute of Psychology, Albert-Ludwigs University Freiburg, Freiburg, Germany; ^3^ Medical Psychology and Medical Sociology, Faculty of Medicine, Albert-Ludwigs University Freiburg, Freiburg, Germany

**Keywords:** smart sensing, digital health, acceptance, implementation, unified theory of acceptance and use of technology acceptance of smart sensing

## Abstract

**Background:**

Accurate and timely diagnostics are essential for effective mental healthcare. Given a resource- and time-limited mental healthcare system, novel digital and scalable diagnostic approaches such as smart sensing, which utilizes digital markers collected via sensors from digital devices, are explored. While the predictive accuracy of smart sensing is promising, its acceptance remains unclear. Based on the unified theory of acceptance and use of technology, the present study investigated (1) the effectiveness of an acceptance facilitating intervention (AFI), (2) the determinants of acceptance, and (3) the acceptance of adults toward smart sensing.

**Methods:**

The participants (*N* = 202) were randomly assigned to a control group (CG) or intervention group (IG). The IG received a video AFI on smart sensing, and the CG a video on mindfulness. A reliable online questionnaire was used to assess acceptance, performance expectancy, effort expectancy, facilitating conditions, social influence, and trust. The self-reported interest in using and the installation of a smart sensing app were assessed as behavioral outcomes. The intervention effects were investigated in acceptance using *t*-tests for observed data and latent structural equation modeling (SEM) with full information maximum likelihood to handle missing data. The behavioral outcomes were analyzed with logistic regression. The determinants of acceptance were analyzed with SEM. The root mean square error of approximation (RMSEA) and standardized root mean square residual (SRMR) were used to evaluate the model fit.

**Results:**

The intervention did not affect the acceptance (*p* = 0.357), interest (OR = 0.75, 95% CI: 0.42–1.32, *p* = 0.314), or installation rate (OR = 0.29, 95% CI: 0.01–2.35, *p* = 0.294). The performance expectancy (*γ* = 0.45, *p* < 0.001), trust (*γ* = 0.24, *p* = 0.002), and social influence (*γ* = 0.32, *p* = 0.008) were identified as the core determinants of acceptance explaining 68% of its variance. The SEM model fit was excellent (RMSEA = 0.06, SRMR = 0.05). The overall acceptance was *M* = 10.9 (SD = 3.73), with 35.41% of the participants showing a low, 47.92% a moderate, and 10.41% a high acceptance.

**Discussion:**

The present AFI was not effective. The low to moderate acceptance of smart sensing poses a major barrier to its implementation. The performance expectancy, social influence, and trust should be targeted as the core factors of acceptance. Further studies are needed to identify effective ways to foster the acceptance of smart sensing and to develop successful implementation strategies.

**Clinical Trial Registration:**

identifier 10.17605/OSF.IO/GJTPH.

## 1. Introduction

Mental disorders are rising in prevalence worldwide (1–3) and constitute a leading cause of years lived with disability (4) and economic costs (5, 6). Effective treatment options exist ranging from face-to-face treatment (e.g., cognitive behavioral therapy) (7, 8) and pharmacological treatment (9, 10) to digital mental health interventions (11, 12). However, a fundamental prerequisite to treatment is an accurate diagnosis and the identification of clinically relevant symptoms (13–15). Facing economic pressure and limited resources in many healthcare systems (16–18), researchers develop novel digital diagnostic procedures such as smart sensing aiming for a scalable, accurate, and time-efficient diagnosis (19–22).

In the context of mental health diagnoses, smart sensing is used to predict mental disorders and mental symptoms by features generated based on digital markers collected via the smartphone or other wearables (e.g., time stayed at home derived from the GPS sensor) (23, 24). Recent studies show the high potential of smart sensing (20, 21, 25–31). For instance, depression status could be detected with over 90% accuracy solely based on smartphone data (21). But also, in other mental disorders (e.g., psychosis spectrum or bipolar disorder), smart sensing achieves promising results (30–35).

However, before applying novel diagnostic approaches such as smart sensing into clinical routine care, it is important to assess the acceptance of it and identify factors associated with using smart sensing. The unified theory of acceptance and use of technology (UTAUT) (36) is a widely applied framework for the use and acceptance of technology (37, 38). UTAUT analyzed several behavior change models to identify *performance expectancy* as the perception of personal benefit derived from utilizing the technology, *effort expectancy* as the anticipated ease of use, *social influence* as the perception that others consider the technology worthwhile, and *facilitating conditions* as the expected support and availability of practical resources as the core determinants of acceptance (36, 37). Given the successful validation of the UTAUT in various contexts [e.g., Internet finance (39), electronic health records (40), or digital health interventions (38)], it may also provide a strong framework to investigate the acceptance of smart sensing and its determinants. In addition, trust has been identified as an important factor influencing the acceptance in application areas of technology and AI-augmented systems [e.g., automatic driving (41)]. Based on the trust concept in automated technology (42), we define trust in smart sensing as the attitude that a smart sensing system can help achieve an individual's goal in an uncertain or vulnerable situation. For instance, a smart sensing system for mental health could prompt a user that they show a high risk for depression and recommend action (e.g., changing routines or visiting a therapist). The users could either show trust in the system (e.g., following the action recommendations) or distrust the system (e.g., rejecting the recommendations). Similar to the findings in automatic driving [e.g., (43)], trust might be affecting the acceptance of smart sensing. However, the role of trust in the acceptance of smart sensing has not been examined.

Besides, understanding the determinants of acceptance of smart sensing, opportunities to foster the acceptance of smart sensing need to be explored for a successful implementation of smart sensing. In the past, *acceptance facilitating interventions* (AFIs) have been shown to be effective in influencing the acceptance of novel approaches (e.g., Internet-based or blended psychotherapy) (44–48). AFIs are usually grounded on an acceptance model such as UTAUT (36, 37) or other models [e.g., health action process approach (49)]. Based on the underlying theoretical background (e.g., UTAUT), assumed determinants of acceptance are directly targeted to increase the acceptance: for instance, in an AFI constructed based on UTAUT, the performance expectancy could be targeted by highlighting the personal benefit, effort expectancy by showing the novel approach in action, social influence by providing reports of other users, and facilitating conditions by targeting concerns of practical resources or the availability of support. In addition to their background, AFI can be characterized by their presentation formats (e.g., written informative text, expert talks, videos, or one-on-one conversations). However, while AFIs were explored in various settings (44–48), it is unknown whether they are effective in the context of smart sensing.

Given the success of the UTAUT model in the context of technology and its application in AFI in other contexts [e.g., Internet- and mobile-based interventions (38)], the present study examines the effectiveness of an UTAUT-based AFI on the acceptance of smart sensing compared with an attention control group.

1. We hypothesize that (a) the self-reported acceptance, (b) interest in using a smart sensing app, and (c) installation of a smart sensing app will be higher in the intervention group compared with the control group.

In addition, the present study aims to apply the UTAUT framework extended by a trust factor in the context of smart sensing to investigate the core determinants of the acceptance of smart sensing.

2. We hypothesize that the UTAUT factors are determinates of acceptance of smart sensing.

Lastly, the present study investigates the general level acceptance of smart sensing (i.e., unmanipulated acceptance in the control group) and answers the following question:

3. What is the acceptance of smart sensing (i.e., self-reported acceptance, interest in using a smart sensing app, installation of a smart sensing app) in the context of mental health?

## 2. Methods and materials

### 2.1. Study design and sample

We report on a randomized controlled trial with a single post-assessment to investigate the effect of the AFI between an intervention group (IG) and a control group (CG). The participants were allocated to IG or CG using a 1:1 randomization. The randomization was conducted automatically by the online survey platform LimeSurvey. The allocation sequence was concealed for the participants and trial personnel until the participants were enrolled and assigned to the groups. All procedures were approved by the ethics committee of Ulm University (462/20—FSt/Sta) and registered at OSF (10.17605/OSF.IO/GJTPH). The registration took place after all the assessment procedures were finalized and recruitment had started. The data were not accessed or analyzed before registration.

The sample size planning assumed that the AFI affects the acceptance, and the increase in acceptance carries over to an increased usage of a smart sensing app (installation of a smart sensing application: yes/no; see measures and outcomes). A usage rate of 20% in the CG and 33% in the IG was expected. To detect this effect using logistic regression with a power of 80% and an *α* = 5%, an effective sample of *N* = 124 was needed. However, due to a technical limitation, the smart sensing app was only functional on Android devices. To avoid potential bias in the investigation of acceptance and determinates of acceptance by excluding users of other systems (e.g., iOS), the possession of an Android smartphone was not defined as an inclusion criterion. Instead, the recruitment was continued until a maximum of *N* = 206 participants to adjust for the forced dropout of Apple iPhone users (or other operating systems).

### 2.2. Inclusion criteria and data collection procedures

All procedures and data collection were conducted online. Aiming to recruit participants from the general population, the participants were recruited and forwarded to the online survey via digital (e.g., email lists, social media posts) and analog (e.g., flyers, on-site recruitment) ways from April 2021 to June 2021. The flyers and on-site recruitment included public (e.g., libraries, fitness centers) and university-related places at Ulm and Freiburg in Germany.

Inclusion criteria were as follows: (1) being of legal age (≥18 years), (2) having Internet access, (3) providing informed consent, and (4) agreement to data processing procedures according to the European General Data Protection Regulation. The online survey was aborted if the criteria were not fulfilled. The eligible participants first answered socio-demographic and mental health questionnaires followed by their automatic randomization to one of two videos (IG or CG; see description below). The participants were not explicitly informed about their group allocation. However, they were aware of the presence of two different conditions due to the informed consent process. After the video, the acceptance of smart sensing and the assumed determinants were assessed (see outcomes below). In addition, the participants could sign up for a study, in which they could use a smart sensing app. The sign-up process did not include any intervention content. The participants received only the information that the university is conducting a smart sensing study without any further explanation of smart sensing or, e.g., how they could benefit from using the smart sensing app. The participants were prompted to indicate whether they are interested in participating in that smart sensing study. The participants reporting interest were forwarded to a survey page, where they could provide their email to be invited to the smart sensing study. The information about expense allowance in the smart sensing study was included on the forwarded survey page. This process was the same in IG and CG.

After completion of this acceptance study, the participants who were psychology students at Ulm University and the University of Freiburg could receive credits for their course of studies, and all the participants could participate in a lottery for one 20 Euro voucher.

### 2.3. Intervention and control condition

The experimental intervention was a video with a total duration of 3:04 min. The structure and content of the video were based on the UTAUT model and focused on the assumed determinants of acceptance: performance expectancy (e.g., presenting application areas such as early recognition of mental health symptoms), effort expectancy (information on effort: e.g., data are mainly collected passively without additional effort for the user), facilitating conditions (information on needed resources: e.g., the broad availability of smartphones), and social influence (e.g., the inclusion of user reports and why others think smart sensing is use-worthy). First, an expert (YT) explained the concept of smart sensing and application areas in healthcare. The expert talk was structured in the following parts: (1) “What is smart sensing?” (2) “Which data is collected?” and (3) “Aims and application areas.” Afterward, three examples were presented of how smart sensing applications could be used in daily life and which benefit it provides for the users. The examples focused on (a) sleep monitoring, (b) physical activity, and (c) general wellbeing. A summary of the key concepts and examples presented in the AFI can be found in Supplementary Material 1.

In the control condition, the participants received a video with an expert (EM) explaining the concept of mindfulness, its influence on health, and suggestions on how mindfulness can be integrated into daily life (e.g., meditation exercises). The total duration of the control video was 3:00 min.

### 2.4. Measures and outcomes

#### 2.4.1. Participant characteristics

We assessed the age, gender, nationality, and personality to describe the participant characteristics. Personality assessment was conducted using the 10-item version of the Big Five Inventory [BFI-10; (50)]. The BFI-10 assesses openness, conscientiousness, extraversion, agreeableness, and neuroticism with a 5-point Likert scale from “fully disagree” to “fully agree.” The BFI-10 shows good reliability and validity (50). In addition, the eight-item version of the patient health questionnaire (PHQ-8) and the seven-item version of the generalized anxiety disorder questionnaire (GAD-7) were used for a reliable assessment of depression (PHQ-8) and anxiety (GAD-7) symptoms in the last 2 weeks (51, 52). The items (e.g., feeling nervous, anxious, or on the edge) were answered from 0—“not at all” to 3—“nearly every day.” According to their scoring procedures, the sum scores for PHQ-8 and GAD-7 were calculated.

#### 2.4.2. Acceptance

Acceptance was operationalized in three ways. First, it was assessed as a continuous dimension with the UTAUT questionnaire (36–38) consisting of four items rating the intention to use smart sensing on a 5-point Likert scale ranging from “fully disagree” to “fully agree” (=self-reported acceptance). The items are presented in Supplementary Material 2. Second, it was determined by the number and percentage of the participants registering for the study (=interest), in which they could use a smart sensing app, and third the actual number and percentage of installation of the smart sensing app were assessed as a direct behavioral outcome.

#### 2.4.3. Potential determinants of acceptance

The performance expectancy (three items), effort expectancy (three items), social influence (two items), and facilitating conditions (two items) were assessed as potential determinants of acceptance with the UTAUT questionnaire (36–38). All items were rated on a 5-point Likert scale from “fully disagree” to “fully agree.” The items are presented in Supplementary Material 2.

In addition, trust (e.g., trust in smart sensing-based treatment recommendations) was assessed with the short version of the German automation trust scale (41, 53). The scale was originally developed in the context of automated driving and adapted to the context of digital health. It consists of seven items (e.g., “I trust the system”) rated on a 7-point Likert scale from “fully disagree” to “fully agree.”

### 2.5. Analysis

#### 2.5.1. Intervention effects

Intervention effects were operationalized on three levels: (1) the self-reported acceptance in the UTAUT questionnaire, (2) the reported interest rate to use a smart sensing app, and (3) the installation of a smart sensing app.

The self-reported acceptance of smart sensing was analyzed by investigating the mean difference between IG and CG in the observed data using an unpaired *t*-test. In addition, we investigated the intervention effect on acceptance following the intention to treat principle. Therefore, we applied the structural equation modeling (SEM). First, a measurement model was defined in SEM analysis consisting of the latent factors for all items of acceptance, performance expectancy, effort expectancy, facilitating conditions, social influence, and trust. In all SEM analyses, the root mean square error of approximation (RMSEA) as a non-centrality parameter and the standardized root mean square residuals (SRMR) as a residual index were used to assess the goodness of fit due to the tendency of the *χ*^2^-test to reject the misspecified models too harshly (54–56). Following the established guidelines, a cut-off value of RMSEA ≤ .06 and SRMR ≤ .08 were chosen to determine a good model fit (57). The full information maximum likelihood was used to handle missing data (58). Robust (Huber–White) standard errors were obtained. In the second step of SEM analysis, a regression from the acceptance factor to the group variable (dummy coded: CG = 0, IG = 1) was introduced. The path loading of the dummy coded group variable on the latent acceptance factor was the parameter of interest to determine the effects of the intervention on latent level.

The intervention effects on the interest rates (dummy coded outcome: 0: not interested, 1: interested) were investigated with a logistic regression model. Odds ratio were reported as effect sizes. Analog potential differences in the installation rate of a smart sensing app were analyzed.

#### 2.5.2. Latent structural equation modeling: determinants of acceptance

To investigate the influence of potential determinants of acceptance, SEM was applied. Building on the measurement model consisting of the latent factors for all items of acceptance, performance expectancy, effort expectancy, facilitating conditions, social influence, and trust, we introduced paths between acceptance and all other latent factors. These path estimates were used to determine the presence of significant effects of the postulated UTAUT factors on acceptance (see Section 2.5.1 for SEM criteria and process).

#### 2.5.3. Acceptance of smart sensing for health

Following the previous studies on the acceptance of digital interventions (44–48), the acceptance (i.e., self-reported, interest rates, and installation rates) in the CG that did not receive any AFI is assumed to be the general acceptance of smart sensing. Acceptance is quantified by the mean and standard deviation of the sum score of the UTAUT questionnaire (numerical mean of the scale: 12.5, range: 4–20). In addition, we categorized the sum score following the previous studies (44–48): low acceptance (sum score: 4–9), moderate acceptance (sum score: 10–15), and high acceptance (sum score: 16–20). The percentages for each category were summarized.

### 2.6. Software

The statistical software R was used for all analyses (59). The R package “lavaan” was used as the core package for all the structural equation models (60). See Supplementary Material 3 for an overview of all packages and versions used in the present analysis.

## 3. Results

**Figure 1:**
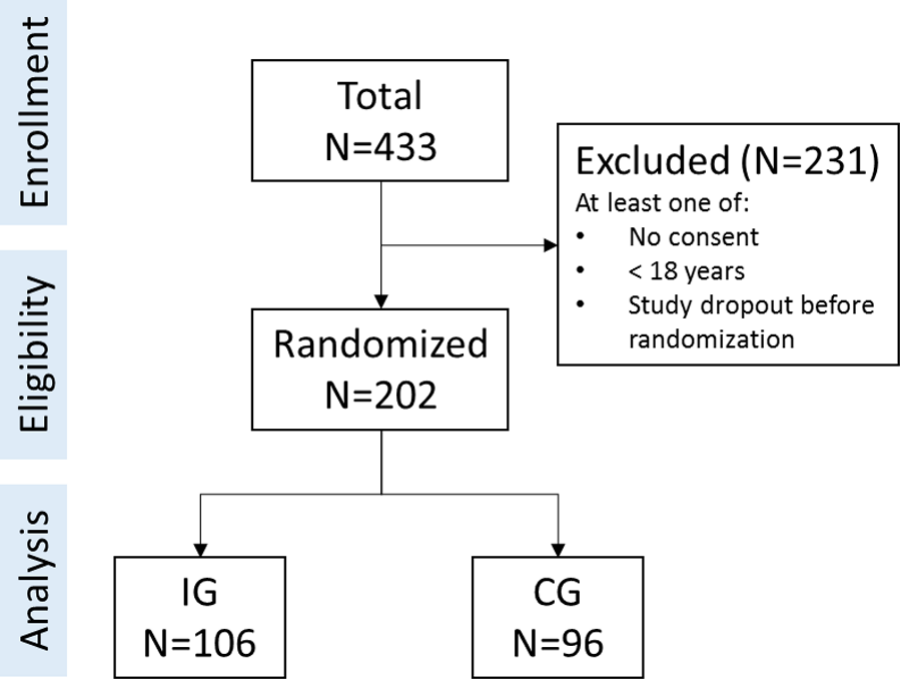
Study flow.

Of *N* = 433 interested individuals, a total of *N* = 202 were eligible and included in the study (CG: *n* = 96; IG: *n* = 106). The study flow is summarized in Figure 1. The included participants covered a broad age range from 18 years to 79 years (*M* = 30.77, SD = 15.82). Gender was unequally represented in the study (female: *n* = 157, 77.72%). All participants had a European background with the majority being German (*n* = 186, 92.08%). Education level was distributed as follows: advanced education level *n* = 65 [32.18%; e.g., bachelor degree and higher or other International Standard Classification of Education (ISCED-11) level >4 qualifications], intermediate education level *n* = 123 (60.89%; e.g., A levels, completed an apprenticeship or other ISCED-11 level <5 qualifications), and basic education level *n* = 13 (6.44%; i.e., no qualification or other ISCED-11 level <3 qualifications; one participant did not report on qualification level). Mental health symptoms were below clinical relevance on average (PHQ-8: *M* = 6.17, SD = 4.05; GAD-7: *M* = 5.25, SD = 4.22). Baseline differences did not suggest a problem with the randomization process. For group-specific details, see Table 1.

**Table 1:** Descriptive sample characteristics.

	All	IG	CG
	*N* = 202	*n* = 106	*n* = 96
Age	*M* = 30.77	*M* = 31.11	*M* = 30.39
	SD = 15.82	SD = 15.59	SD = 16.14
Gender (female)	*n* = 157 (77.72%)	*n* = 82 (77.36%)	*n* = 75 (78%)
Personality facets			
Openness	*M* = 3.75	*M* = 3.71	*M* = 3.79
	SD = 0.99	SD = 1.03	SD = 0.95
Conscientiousness	*M* = 3.74	*M* = 3.76	*M* = 3.72
	SD = 0.82	SD = 0.82	SD = 0.82
Extraversion	*M* = 3.47	*M* = 3.53	*M* = 3.42
	SD = 0.94	SD = 0.97	SD = 0.91
Agreeableness	*M* = 3.38	*M* = 3.44	*M* = 3.32
	SD = 0.79	SD = 0.72	SD = 0.85
Neuroticism	*M* = 3.15	*M* = 3.12	*M* = 3.19
	SD = 0.99	SD = 0.99	SD = 1.00
Nationality			
German	*n* = 186 (92.08%)	*n* = 97 (91.51%)	*n* = 89 (92.71%)
Other European	*n* = 16 (7.92%)	*n* = 9 (8.49%)	*n* = 7 (7.29%)
Qualification level^a^			
Basic	*n* = 13 (6.44%)	*n* = 4 (3.77%)	*n* = 9 (9.38%)
Intermediate	*n* = 123 (60.89%)	*n* = 67 (63.21%)	*n* = 56 (58.33%)
Advanced	*n* = 65 (32.18%)	*n* = 34 (32.08%)	*n* = 31 (32.29%)
Depression (PHQ-8)	*M* = 6.17	*M* = 5.85	*M* = 6.51
	SD = 4.05	SD = 3.78	SD = 4.32
Anxiety (GAD-7)	*M* = 5.25	*M* = 5.14	*M* = 5.37
	SD = 4.22	SD = 4.21	SD = 4.25

^a^ Education level is summarized according to the International Standard Classification of Education: ISCED-11.

### 3.1. Intervention effects

With an average self-reported acceptance of *M* = 11.42 (SD = 4.07, Min = 4, Max = 19) in the IG, there was descriptively higher acceptance compared with the control group, but with no significant difference (*d* = 0.14, 95% CI: −0.15–0.43; *t* = 0.92, df = 187, *p* = 0.357). This held true on the latent level using the SEM and accounting for missingness (*γ* = 0.11, 95% CI: −0.21–0.43, *p* = 0.503; *γ*_standardized _= 0.05). The model fit for the underlying questionnaire was excellent (RMSEA = 0.06, SRMR = 0.05). The full parameter list of the measurement model is reported in Supplementary Material 4.

With interest rates of *n*_yes _= 39 (36.79%) and *n*_no _= 57 (53.77%) in the IG, the odds for being interested to use a smart sensing app did not differ significantly compared with the CG (OR = 0.75, 95% CI: 0.42–1.32, *p* = .314). Only one participant (0.94%) in the IG installed the smart sensing app, amounting to a non-significant intervention effect compared with the CG (OR = 0.29, 95% CI: 0.01–2.35, *p* = .294). For a summary of all intervention effects and group-specific results see Table 2.

**Table 2:** Summary of intervention effects.

Outcome	CG	IG	Effect size	CI	*p*
Acceptance	*M* = 10.90	*M* = 11.42	*d* = 0.14^a^	−0.15 to 0.43	0.357
	SD = 3.73	SD = 4.07	*γ* = 0.11^b^	−0.21 to 0.43	0.504
Interest	*n* = 42 (43.75%)	*n* = 39 (36.79%)	OR = 0.75	0.42 to 1.32	0.314
Installation	*n* = 3 (3.13%	*n* = 1 (0.94%)	OR = 0.29	0.01 to 2.35	0.294

^a^ Mean difference between IG and CG based on observed data.

^b^ Unstandardized group difference between IG and CG based on SEM.

### 3.2. Determinants of acceptance

**Figure 2:**
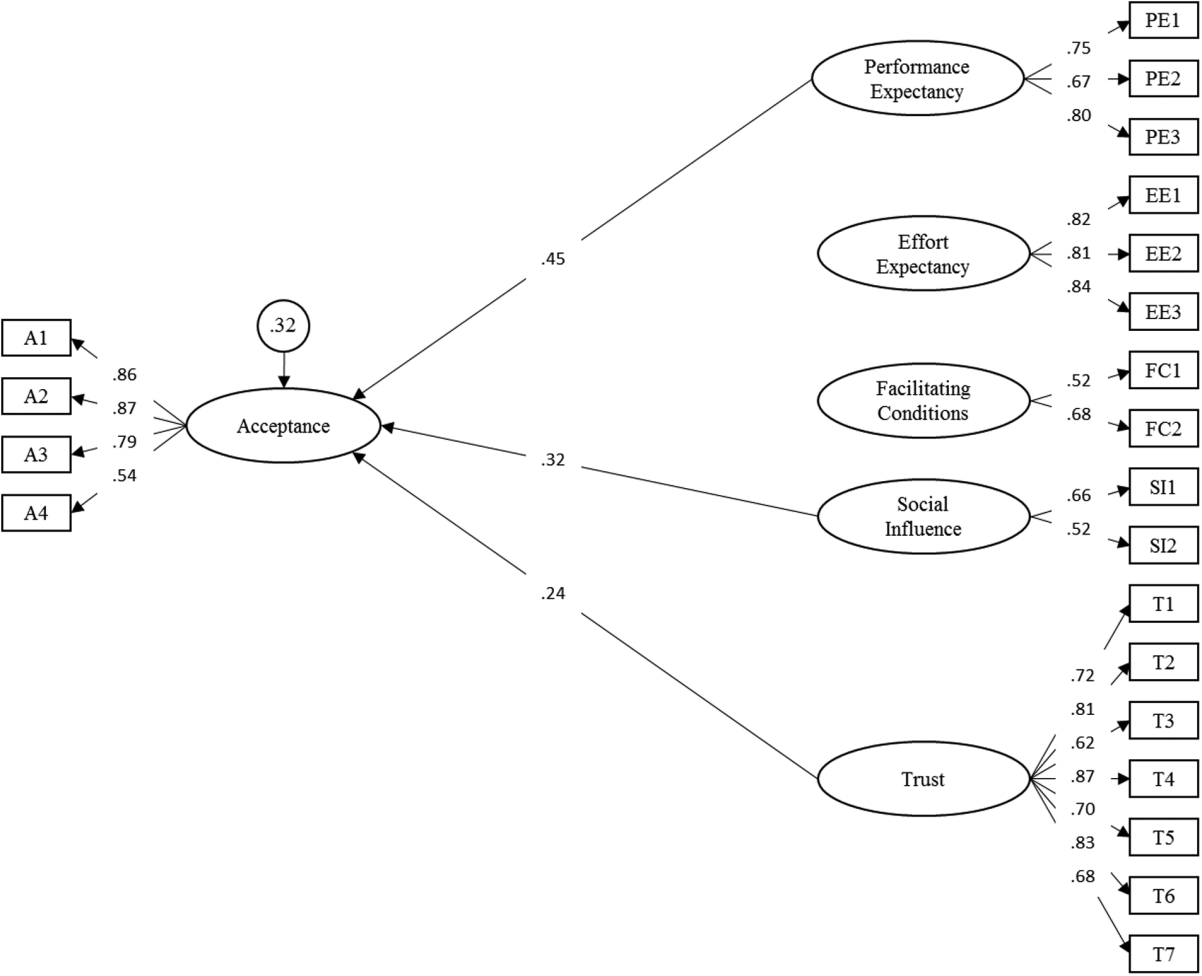
Structural equation model of adapted model for the acceptance toward smart sensing. Latent variables are represented in ellipses: A, acceptance; PE, performance expectancy; EE, effort expectancy; FC, facilitating conditions; SI, social influence; T, trust. Observed items are indicated as rectangles. Path loadings are represented as single-headed arrows. Residual variances of endogenous latent variables are presented in circles. All exogenous latent variables were allowed to correlate. For improved readability, all latent correlations and residual variances of manifest items were omitted. Please see Supplementary Material 3 for a full list of all parameters.

The following analysis of the latent effects on acceptance across groups identified the performance expectancy (*γ* = 0.45, *p* < 0.001), trust (*γ* = 0.24, *p* = 0.002), and social influence (*γ* = 0.32, *p* = 0.008) as determinants of acceptance (overall model fit: RMSEA = 0.06, SRMR = 0.05). All other factors were non-significant. Together, the three determinants explained 68% of the variance of the latent acceptance factor. The final path model is displayed in Figure 2. A list of all parameters is included in Supplementary Material 5.

### 3.3. General acceptance of smart sensing

**Figure 3:**
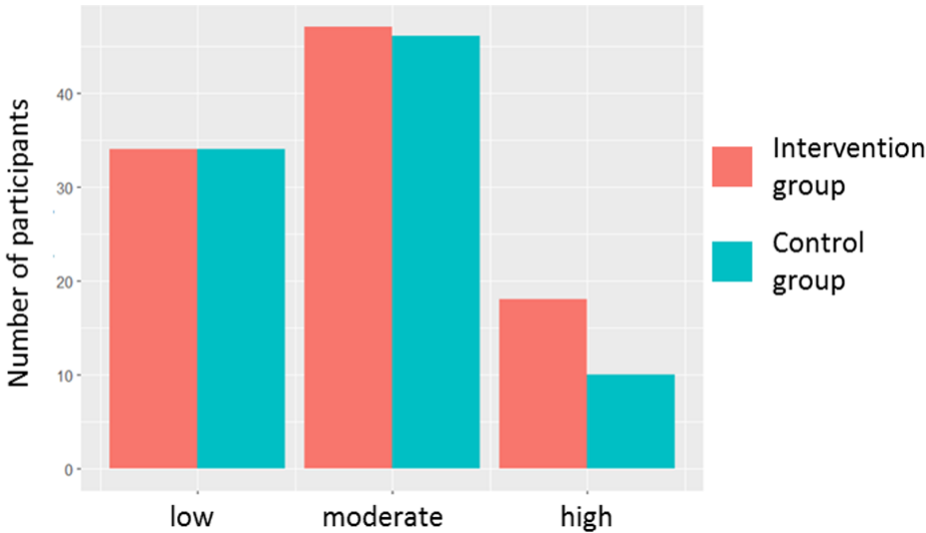
Acceptance of smart sensing. Acceptance was measured by the UTAUT questionnaire. The sum score of all four acceptance items was categorized as low (sum score: 4–9), moderate (sum score: 10–15), and high (sum score: 16–20).

The unmanipulated self-reported acceptance of smart sensing in the control group was below average *M* = 10.90 (SD = 3.73, Min = 4, Max = 20). A total of *n* = 34 (35.41%) showed low, *n* = 46 (47.92%) moderate, and *n* = 10 (10.41%) high acceptance (see Figure 3). For a descriptive summary of the acceptance and subscales, please see Supplementary Material 6.

A total of *n* = 42 (43.75%) participants stated interest to try smart sensing in another study (no interest: *n* = 45, 46.86%; not responded: *n* = 9, 9.38%). Of all 42 participants with interest, only *n* = 3 (7.14%; 3.13% of all participants in the CG) installed the smart sensing app.

## 4. Discussion

Acceptance is a fundamental precondition for the dissemination, uptake, and clinical impact. The present UTAUT-based acceptance facilitating intervention was unable to significantly affect the acceptance of smart sensing. We identified three core determinants for the acceptance of smart sensing measured with the UTAUT questionnaire: combined the performance expectancy, the social influence, and the trust factor explained 68% of the variance in the self-reported acceptance. Most participants reported a below-average acceptance toward smart sensing in the UTAUT questionnaire and even one-third showed low acceptance, highlighting the sensitivity of smart sensing. Interestingly, despite the low acceptance in the questionnaire, 44% stated general interest in using smart sensing. Though, the actual installation rate of a smart sensing app as a behavioral outcome was below 5%.

The lack of acceptance of smart sensing by potential end users clearly highlights a major barrier to the implementation of smart sensing applications. Developing successful AFI and implementation strategies are of utmost importance to fully exploit the potential of smart sensing. As shown in the structural equation modeling, the performance expectancy had the strongest influence on self-reported acceptance with an effect of *γ* = 0.45. Accordingly, the personal benefit for individuals should especially be highlighted, when the intention of the individual to use a smart sensing application is targeted.

However, besides the context of AFI, future studies should explore which formats (e.g., with extended case examples in daily life, a showcase of specific example apps and functions, or an in-person AFI with direct participant interaction) are best suited to achieve positive effects. For instance, the present AFI format consisting of a combination of an expert talk with short examples was unable to achieve this goal despite targeting the determinants postulated in UTAUT. Looking to the field of instructional design and online learning research, in particular, whiteboard videos may pose an opportunity to development scalable and effective AFI (61). Following the cognitive theory of multimedia learning, information (e.g., on the personal benefit) could be divided in verbal and visual components to reduce cognitive load during the intervention (62). Furthermore, a dynamic visualization of content and narrative style could potentially increase the effectiveness of whiteboard-based AFI (61, 63, 64). However, the effectiveness of such AFI in the context of smart sensing is currently unclear and needs to be explored.

Extending the findings of below-average acceptance in the questionnaire data, the transfer from behavioral intention to use smart sensing to the usage of smart sensing was identified as another issue in the present study: even in the subset of the participants stating interest in using a smart sensing app, only 7% installed the smart sensing app (3% if participants stating no interest are also included). Due to the low usage rate of the smart sensing app, the present study allowed no robust analysis of the factors influencing the relationship between intention to use and actual usage. Future studies on the use of smart sensing and how the transfer from intention to action can be maximized are needed. Based on another study in the context of mobile health, the factors such as existing habits and personal empowerment might be promising variables to investigate (65). In addition, it must be highlighted that installing a smart sensing app marks only the starting point of actual usage. Future studies should extend on this by observing not only the start of usage but also monitoring the duration, frequency of the use, and retention time (i.e., days past until an app is no longer opened) of a smart sensing app over time (66). The long-term use of digital applications has been identified as a major issue in the previous studies (11, 48, 66–69). Particularly in smart sensing, which is usually implemented as a longitudinal process requiring assessment over a longer period, early dropout could have a major impact on its potential benefit in healthcare. Hence, approaches fostering the maintenance of adherence and the prevention of disengagement over time need to be explored. For instance, therapeutic persuasiveness, user engagement, and usability may be important factors based on the findings in eHealth interventions (68, 70). Overall, the promising findings of smart sensing (30–35) will only translate into healthcare improvements if the requirement of acceptance is met and the underlying processes for the initial and long-term usage are understood.

While speaking of implications for future studies as well as when interpreting the present results, some limitations of the present study should be considered: the present sample showed an imbalance in education level, national backgrounds (i.e., >90% German), and gender (78% female). In addition, although a broad age range (18–79 years) was included, children and adolescents were excluded from this study. Given the higher usage of smartphones and affinity to digital platforms and technologies in younger individuals (71), the acceptance of smart sensing might be different in that population. Furthermore, the recruitment was not conducted in a clinical setting. As a result, depression (PHQ-8) and anxiety (GAD-7) symptoms were in a sub-clinical range. Since the perceived personal benefit was identified as the most important predictor, the general acceptance might be higher in a clinical population in which the benefits of smart sensing and tracking of health symptoms or diagnosis are more apparent (e.g., symptom tracking, early warning of relapse risk). Hence, generalizations of the below-average acceptance to the mental healthcare sector should be made carefully, and additional studies on the acceptance in patients and other stakeholders (e.g., psychotherapists) are required.

Furthermore, the present study assessed the acceptance of smart sensing with no further differentiation between data types (e.g., smartphone usage time or GPS data) or the recipients of data (e.g., physicians). Previous research has shown that the data type and recipients can be influential regarding acceptance (72). Hence, the acceptance of smart sensing in different settings such as tracking physical mobility after surgery or tracking mental health after inpatient psychotherapy might differ. Moreover, the degree of autonomous agency of a smart sensing system can be varied from (1) a full user-controlled self-monitoring system over (2) a system integrated into expert systems to support clinicians in their decisions to (3) a fully automated diagnosis and treatment system (72, 73). The influence of the varying degree of autonomous agency on the acceptance was not in the scope of the present study and should be examined in future studies.

## 5. Conclusions

The present AFI was unable to significantly impact the acceptance of smart sensing. However, we identified the performance expectancy, social influence, and trust toward smart sensing applications as the key predictors of acceptance. Future studies should focus on these factors and investigate different formats (e.g., whiteboard-based AFI) to improve the acceptance of smart sensing. Moreover, exploring the acceptance of smart sensing in patients and other stakeholders and agents in the mental health sector would be a valuable addition to this study. Based on the low to moderate acceptance of smart sensing found in the present study, the acceptance seems to pose a major barrier for the implementation of smart sensing and its impact. The development of successful implementation strategies including the facilitating of acceptance are highly needed to fully exploit the potential of smart sensing.

## Data Availability

Data requests should be directed to the corresponding author (YT). Data can be shared with researchers who provide a methodologically sound proposal, which is not already covered by other researchers. Data can only be shared for projects if the General Data Protection Regulation is met. Requestors may need to sign additional data access agreements. Support depends on available resources.
