# Supplementary material for: Acceptance of smart sensing: a barrier to implementation—results from a randomized controlled trial
Source: Front Digit Health. 2023 Jul 13;5:1075266. doi: 10.3389/fdgth.2023.1075266 (PMC10373890; doi:10.3389/fdgth.2023.1075266)
Supplement: Supplementary file 1 [file Table1.docx]

Supplementary Material

# Supplement 1 - Summary of AFI components to target UTAUT factors.

| UTAUT factor | key concepts and examples in the AFI |
| --- | --- |
| performance expectancy (i.e. perceived personal benefit) | high burden by mental diseases and potential of smart sensing in assisted diagnosis, monitoring, early-recognition, personalized therapy;  three user reports and how they benefit:   - Sleep monitoring, insights into sleep routine, and improved sleep by self-awareness - Monitoring of physical activity and assisted achievement of personal activity goals (e.g., steps) - Monitoring of smartphone usage and step-count for general well-being |
| effort expectancy (i.e., expected ease of use) | passive collection of data by sensors,  optional integration of short active user inputs (i.e., short daily questionnaires),  comparisons, and similarity to other frequently used applications (e.g., shopping, finance). |
| social influence (i.e., perception others perceive the technology as use-worthy) | Presentation of why others think smart sensing is use-worthy:   - Information/examples provided by the researcher/expert - three reports by users |
| facilitating conditions (e.g., practical resources) | broad availability of smartphones and the necessary technology; |

*Note: Due to copyright and agreements with the persons shown in the videos, the AFI is not publicly available. The here provided list provides a summary of the key concepts included in the acceptance facilitating intervention. More detailed information can be requested by the corresponding author.*

# Supplement 2 - UTAUT items adapted to smart sensing.

| Variable | Items |
| --- | --- |
| Behavioral Intention | 1. I could imagine using a smart sensing app. 2. If offered, I would use a smart sensing app regularly 3. I would recommend a smart sensing app to a friend 4. I would be willing to pay for a smart sensing app |
| Performance Expectancy | 1. Using a smart sensing app could have positive effects on my health. 2. Using a smart sensing app and the collected data could help doctors in diagnoses 3. Overall, a smart sensing app could help managing health. |
| Effort  Expectancy | 1. Using a smart sensing app would be simple 2. Using a smart sensing app would be an easy task for me 3. A smart sensing app would be clear and easily comprehensible to me |
| Social  Influence | 1. People close to me would recommend me to use a smart sensing app 2. My general practitioner would recommend me to use a smart sensing app |
| Facilitating Conditions | 1. I have all necessary technical preconditions for using a smart sensing app 2. In case of technical problems with a smart sensing app I would receive technical support |
| Internet  Anxiety | 1. Smart sensing has something threatening for me 2. I am afraid of making an irrevocable mistake while using smart sensing 3. Using a smart sensing app worries me a lot. |
| *Note.* All items were presented in German and all present model fits refer to the German version of the questionnaire. Generalization to the here provided English items in pending. The German items can be requested from the corresponding author. | |

Items were adapted to smart sensing from: Philippi P, Baumeister H, Apolinário-Hagen J, Ebert DD, Hennemann S, Kott L, et al. Acceptance towards digital health interventions – Model validation and further development of the Unified Theory of Acceptance and Use of Technology. Internet Interv 2021;26:100459. https://doi.org/10.1016/j.invent.2021.100459.

# Supplement 3 - R packages and versions

In the present study all analyses have been conducted in R. For an overview of all used packages and their respective versions please see below.


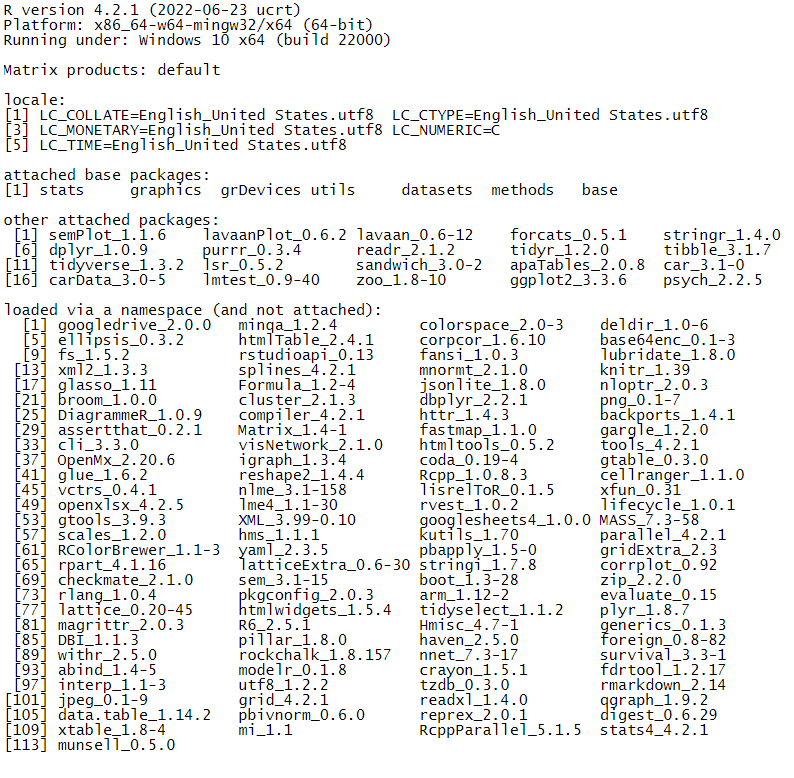


# *Supplement 4 – Measurement model & reliability*

A full list of all parameters in the measurement model (latent variables, covariances, and variances) can be found below. Acceptance is abbreviated (BI), performance expectancy (PE), effort expectancy (EE), facilitating conditions (FC), social influence (SI).

Modelfit: RMSEA=0.056, SRMR=0.047

Parameter Estimates:

Standard errors Sandwich

Information bread Observed

Observed information based on Hessian

Latent Variables:

Estimate Std.Err z-value P(>|z|) Std.lv Std.all

BI =~

UTAUT_BI01_r 1.000 1.075 0.859

UTAUT_BI02_r 1.026 0.055 18.598 0.000 1.103 0.869

UTAUT_BI03_r 0.807 0.069 11.668 0.000 0.867 0.791

UTAUT_BI04_r 0.539 0.068 7.935 0.000 0.579 0.543

PE =~

UTAUT_PE01_r 1.000 0.679 0.756

UTAUT_PE02_r 0.860 0.126 6.837 0.000 0.584 0.673

UTAUT_PE03_r 1.068 0.118 9.078 0.000 0.725 0.796

EE =~

UTAUT_EE01_r 1.000 0.719 0.822

UTAUT_EE02_r 1.168 0.096 12.156 0.000 0.840 0.813

UTAUT_EE03_r 1.039 0.092 11.346 0.000 0.747 0.842

FC =~

UTAUT_FC01_r 1.000 0.327 0.327

UTAUT_FC02_r 1.680 0.676 2.484 0.013 0.550 0.666

SI =~

UTAUT_SI01_r 1.000 0.603 0.663

UTAUT_SI02_r 0.784 0.191 4.107 0.000 0.473 0.519

TRUST =~

LETRAS_I1_r 1.000 1.125 0.719

LETRAS_I2_r 1.211 0.087 13.847 0.000 1.362 0.814

LETRAS_I3_r 0.888 0.107 8.298 0.000 0.999 0.620

LETRASG_I4 1.169 0.114 10.255 0.000 1.316 0.874

LETRASG_I5 0.867 0.123 7.072 0.000 0.976 0.699

LETRASG_I6 1.117 0.124 8.982 0.000 1.257 0.834

LETRAS_I7_r 1.060 0.110 9.609 0.000 1.192 0.684

Covariances:

Estimate Std.Err z-value P(>|z|) Std.lv Std.all

BI ~~

PE 0.533 0.098 5.446 0.000 0.730 0.730

EE 0.386 0.085 4.565 0.000 0.500 0.500

FC 0.087 0.059 1.491 0.136 0.249 0.249

SI 0.425 0.081 5.256 0.000 0.657 0.657

TRUST 0.700 0.132 5.306 0.000 0.579 0.579

PE ~~

EE 0.307 0.076 4.056 0.000 0.628 0.628

FC 0.080 0.060 1.336 0.182 0.358 0.358

SI 0.214 0.063 3.415 0.001 0.522 0.522

TRUST 0.352 0.090 3.914 0.000 0.460 0.460

EE ~~

FC 0.168 0.083 2.012 0.044 0.713 0.713

SI 0.150 0.061 2.477 0.013 0.347 0.347

TRUST 0.394 0.078 5.053 0.000 0.487 0.487

FC ~~

SI 0.051 0.039 1.301 0.193 0.258 0.258

TRUST 0.105 0.065 1.623 0.104 0.286 0.286

SI ~~

TRUST 0.287 0.081 3.557 0.000 0.423 0.423

Intercepts:

Estimate Std.Err z-value P(>|z|) Std.lv Std.all

.UTAUT_BI01_r 3.307 0.091 36.373 0.000 3.307 2.643

.UTAUT_BI02_r 2.968 0.092 32.246 0.000 2.968 2.339

.UTAUT_BI03_r 2.848 0.080 35.751 0.000 2.848 2.596

.UTAUT_BI04_r 2.034 0.077 26.317 0.000 2.034 1.909

.UTAUT_PE01_r 3.558 0.065 54.397 0.000 3.558 3.963

.UTAUT_PE02_r 3.760 0.063 59.533 0.000 3.760 4.335

.UTAUT_PE03_r 3.606 0.066 54.389 0.000 3.606 3.958

.UTAUT_EE01_r 3.960 0.064 62.178 0.000 3.960 4.528

.UTAUT_EE02_r 3.832 0.075 50.951 0.000 3.832 3.710

.UTAUT_EE03_r 3.933 0.065 60.904 0.000 3.933 4.430

.UTAUT_FC01_r 3.634 0.073 49.857 0.000 3.634 3.626

.UTAUT_FC02_r 4.448 0.060 73.887 0.000 4.448 5.390

.UTAUT_SI01_r 2.860 0.066 43.208 0.000 2.860 3.145

.UTAUT_SI02_r 2.649 0.066 39.990 0.000 2.649 2.908

.LETRAS_I1_r 4.594 0.113 40.670 0.000 4.594 2.935

.LETRAS_I2_r 4.208 0.121 34.846 0.000 4.208 2.515

.LETRAS_I3_r 5.135 0.116 44.162 0.000 5.135 3.187

.LETRASG_I4 3.974 0.109 36.589 0.000 3.974 2.641

.LETRASG_I5 4.276 0.101 42.432 0.000 4.276 3.062

.LETRASG_I6 4.031 0.109 37.076 0.000 4.031 2.676

.LETRAS_I7_r 3.740 0.126 29.737 0.000 3.740 2.146

BI 0.000 0.000 0.000

PE 0.000 0.000 0.000

EE 0.000 0.000 0.000

FC 0.000 0.000 0.000

SI 0.000 0.000 0.000

TRUST 0.000 0.000 0.000

Variances:

Estimate Std.Err z-value P(>|z|) Std.lv Std.all

.UTAUT_BI01_r 0.411 0.069 5.967 0.000 0.411 0.263

.UTAUT_BI02_r 0.394 0.075 5.248 0.000 0.394 0.245

.UTAUT_BI03_r 0.451 0.070 6.457 0.000 0.451 0.375

.UTAUT_BI04_r 0.800 0.094 8.474 0.000 0.800 0.705

.UTAUT_PE01_r 0.345 0.063 5.476 0.000 0.345 0.428

.UTAUT_PE02_r 0.411 0.058 7.041 0.000 0.411 0.547

.UTAUT_PE03_r 0.304 0.056 5.414 0.000 0.304 0.366

.UTAUT_EE01_r 0.247 0.041 6.008 0.000 0.247 0.324

.UTAUT_EE02_r 0.361 0.068 5.272 0.000 0.361 0.338

.UTAUT_EE03_r 0.230 0.043 5.339 0.000 0.230 0.291

.UTAUT_FC01_r 0.897 0.110 8.170 0.000 0.897 0.893

.UTAUT_FC02_r 0.379 0.162 2.334 0.020 0.379 0.556

.UTAUT_SI01_r 0.464 0.086 5.406 0.000 0.464 0.561

.UTAUT_SI02_r 0.607 0.086 7.051 0.000 0.607 0.731

.LETRAS_I1_r 1.184 0.182 6.514 0.000 1.184 0.483

.LETRAS_I2_r 0.945 0.185 5.112 0.000 0.945 0.337

.LETRAS_I3_r 1.598 0.177 9.048 0.000 1.598 0.615

.LETRASG_I4 0.534 0.120 4.470 0.000 0.534 0.236

.LETRASG_I5 0.998 0.173 5.751 0.000 0.998 0.512

.LETRASG_I6 0.690 0.144 4.801 0.000 0.690 0.304

.LETRAS_I7_r 1.614 0.242 6.683 0.000 1.614 0.532

BI 1.155 0.127 9.102 0.000 1.000 1.000

PE 0.461 0.100 4.589 0.000 1.000 1.000

EE 0.517 0.098 5.273 0.000 1.000 1.000

FC 0.107 0.068 1.568 0.117 1.000 1.000

SI 0.363 0.108 3.379 0.001 1.000 1.000

TRUST 1.266 0.252 5.015 0.000 1.000 1.000

While the factor loadings in the measurement model provide direct inference regarding how reliable the items are as an indicator for the underlying constructs, we additionally analyzed omega total as a reliability estimate.

| Construct | Omega total |
| --- | --- |
| Acceptance | 0.87 |
| Performance expectancy | 0.80 |
| Effort expectancy | 0.87 |
| Social influence | NA^1^ |
| Facilitating conditions | NA^1^ |
| Trust | 0.93 |

Note: 1) As the underlying Schmid Leiman transformation to find omega requires three factors to define a solution uniquely, the analysis for scales with two items (i.e., social influence and facilitating conditions) was not applicable. Please see the loadings in the estimates of the measurement for interference regarding the goodness of the items as indicators of the underlying construct.

# Supplement 5 – Parameter estimates

A full list of all parameters (latent variables, regression estimates, covariances, and variances) can be found below. Acceptance is abbreviated (BI), performance expectancy (PE), effort expectancy (EE), facilitating conditions (FC), social influence (SI).

Standard errors Sandwich

Information bread Observed

Observed information based on Hessian

**Latent Variables:**

Estimate Std.Err z-value P(>|z|) Std.lv Std.all

BI =~

UTAUT_BI01_r 1.000 1.076 0.860

UTAUT_BI02_r 1.024 0.055 18.563 0.000 1.102 0.869

UTAUT_BI03_r 0.805 0.069 11.711 0.000 0.866 0.790

UTAUT_BI04_r 0.537 0.068 7.927 0.000 0.578 0.543

PE =~

UTAUT_PE01_r 1.000 0.677 0.754

UTAUT_PE02_r 0.863 0.125 6.907 0.000 0.585 0.674

UTAUT_PE03_r 1.074 0.116 9.227 0.000 0.727 0.798

EE =~

UTAUT_EE01_r 1.000 0.719 0.822

UTAUT_EE02_r 1.168 0.096 12.146 0.000 0.840 0.813

UTAUT_EE03_r 1.040 0.091 11.383 0.000 0.748 0.842

FC =~

UTAUT_FC01_r 1.000 0.321 0.320

UTAUT_FC02_r 1.745 0.689 2.533 0.011 0.560 0.679

SI =~

UTAUT_SI01_r 1.000 0.604 0.665

UTAUT_SI02_r 0.782 0.191 4.099 0.000 0.473 0.519

TRUST =~

LETRAS_I1_r 1.000 1.125 0.719

LETRAS_I2_r 1.211 0.087 13.838 0.000 1.362 0.814

LETRAS_I3_r 0.888 0.107 8.291 0.000 0.999 0.620

LETRASG_I4 1.169 0.114 10.247 0.000 1.316 0.874

LETRASG_I5 0.867 0.123 7.063 0.000 0.976 0.699

LETRASG_I6 1.117 0.124 8.977 0.000 1.257 0.834

LETRAS_I7_r 1.060 0.110 9.612 0.000 1.193 0.685

**Regressions:**

Estimate Std.Err z-value P(>|z|) Std.lv Std.all

BI ~

SI 0.566 0.213 2.655 0.008 0.318 0.318

TRUST 0.225 0.072 3.136 0.002 0.236 0.236

PE 0.722 0.164 4.412 0.000 0.454 0.454

**Covariances:**

Estimate Std.Err z-value P(>|z|) Std.lv Std.all

PE ~~

EE 0.305 0.075 4.087 0.000 0.625 0.625

FC 0.073 0.055 1.341 0.180 0.338 0.338

SI 0.213 0.062 3.421 0.001 0.522 0.522

TRUST 0.351 0.090 3.916 0.000 0.460 0.460

EE ~~

FC 0.162 0.082 1.985 0.047 0.703 0.703

SI 0.149 0.061 2.448 0.014 0.342 0.342

TRUST 0.394 0.078 5.048 0.000 0.486 0.486

FC ~~

SI 0.044 0.036 1.219 0.223 0.227 0.227

TRUST 0.101 0.062 1.616 0.106 0.278 0.278

SI ~~

TRUST 0.287 0.081 3.558 0.000 0.422 0.422

**Intercepts:**

Estimate Std.Err z-value P(>|z|) Std.lv Std.all

.UTAUT_BI01_r 3.307 0.091 36.373 0.000 3.307 2.643

.UTAUT_BI02_r 2.968 0.092 32.247 0.000 2.968 2.339

.UTAUT_BI03_r 2.848 0.080 35.752 0.000 2.848 2.596

.UTAUT_BI04_r 2.034 0.077 26.318 0.000 2.034 1.909

.UTAUT_PE01_r 3.558 0.065 54.397 0.000 3.558 3.963

.UTAUT_PE02_r 3.760 0.063 59.533 0.000 3.760 4.335

.UTAUT_PE03_r 3.606 0.066 54.390 0.000 3.606 3.958

.UTAUT_EE01_r 3.960 0.064 62.179 0.000 3.960 4.528

.UTAUT_EE02_r 3.832 0.075 50.951 0.000 3.832 3.710

.UTAUT_EE03_r 3.933 0.065 60.904 0.000 3.933 4.430

.UTAUT_FC01_r 3.634 0.073 49.854 0.000 3.634 3.627

.UTAUT_FC02_r 4.448 0.060 73.887 0.000 4.448 5.390

.UTAUT_SI01_r 2.860 0.066 43.209 0.000 2.860 3.145

.UTAUT_SI02_r 2.649 0.066 39.992 0.000 2.649 2.908

.LETRAS_I1_r 4.594 0.113 40.670 0.000 4.594 2.935

.LETRAS_I2_r 4.208 0.121 34.846 0.000 4.208 2.515

.LETRAS_I3_r 5.135 0.116 44.162 0.000 5.135 3.187

.LETRASG_I4 3.974 0.109 36.589 0.000 3.974 2.641

.LETRASG_I5 4.276 0.101 42.432 0.000 4.276 3.062

.LETRASG_I6 4.031 0.109 37.076 0.000 4.031 2.676

.LETRAS_I7_r 3.740 0.126 29.737 0.000 3.740 2.146

.BI 0.000 0.000 0.000

PE 0.000 0.000 0.000

EE 0.000 0.000 0.000

FC 0.000 0.000 0.000

SI 0.000 0.000 0.000

TRUST 0.000 0.000 0.000

**Variances:**

Estimate Std.Err z-value P(>|z|) Std.lv Std.all

.UTAUT_BI01_r 0.408 0.069 5.957 0.000 0.408 0.261

.UTAUT_BI02_r 0.395 0.075 5.247 0.000 0.395 0.245

.UTAUT_BI03_r 0.453 0.070 6.512 0.000 0.453 0.376

.UTAUT_BI04_r 0.801 0.094 8.487 0.000 0.801 0.705

.UTAUT_PE01_r 0.348 0.062 5.596 0.000 0.348 0.431

.UTAUT_PE02_r 0.410 0.058 7.063 0.000 0.410 0.545

.UTAUT_PE03_r 0.301 0.057 5.310 0.000 0.301 0.362

.UTAUT_EE01_r 0.248 0.041 6.003 0.000 0.248 0.324

.UTAUT_EE02_r 0.361 0.069 5.272 0.000 0.361 0.339

.UTAUT_EE03_r 0.230 0.043 5.343 0.000 0.230 0.291

.UTAUT_FC01_r 0.901 0.108 8.362 0.000 0.901 0.897

.UTAUT_FC02_r 0.367 0.163 2.258 0.024 0.367 0.539

.UTAUT_SI01_r 0.462 0.086 5.360 0.000 0.462 0.558

.UTAUT_SI02_r 0.607 0.086 7.058 0.000 0.607 0.731

.LETRAS_I1_r 1.184 0.182 6.513 0.000 1.184 0.483

.LETRAS_I2_r 0.944 0.185 5.099 0.000 0.944 0.337

.LETRAS_I3_r 1.598 0.177 9.045 0.000 1.598 0.616

.LETRASG_I4 0.534 0.120 4.466 0.000 0.534 0.236

.LETRASG_I5 0.998 0.174 5.746 0.000 0.998 0.512

.LETRASG_I6 0.690 0.144 4.802 0.000 0.690 0.304

.LETRAS_I7_r 1.614 0.242 6.681 0.000 1.614 0.531

.BI 0.376 0.095 3.961 0.000 0.325 0.325

PE 0.459 0.100 4.596 0.000 1.000 1.000

EE 0.517 0.098 5.272 0.000 1.000 1.000

FC 0.103 0.066 1.570 0.116 1.000 1.000

SI 0.365 0.107 3.405 0.001 1.000 1.000

TRUST 1.266 0.252 5.014 0.000 1.000 1.000

# Supplement 6 – Descriptive summary of acceptance and its determinates

| Subscale | Intervention group  M (SD) | Control group  M (SD) | All  M (SD) |
| --- | --- | --- | --- |
| Acceptance | 11.42 (4.07) | 10.90 (3.73) | 11.17 (3.91) |
| Performance expectancy | 11.06 (2.35) | 10.79 (2.13) | 10.93 (2.25) |
| Effort expectancy | 12.16 (2.40) | 11.27 (2.51) | 11.74 (2.49) |
| Facilitating conditions | 8.20 (1.30) | 7.96 (1.56) | 8.08 (1.43) |
| Social influence | 5.55 (1.35) | 5.48 (1.65) | 5.51 (1.50) |
| Trust | 30.72 (8.88) | 29.13 (8.45) | 29.96 (8.69) |
